# The Effect of the Mixed Extract of *Kalopanax pictus* Nakai and *Achyranthes japonica* Nakai on the Improvement of Degenerative Osteoarthritis through Inflammation Inhibition in the Monosodium Iodoacetate-Induced Mouse Model

**DOI:** 10.3390/cimb45080404

**Published:** 2023-08-01

**Authors:** Hak-Yong Lee, Young-Mi Park, Hai-Min Hwang, Dong-Yeop Shin, Han-Na Jeong, Jae-Gon Kim, Hyo-Yeon Park, Dae-Sung Kim, Jin-Joo Yoo, Myung-Sunny Kim, Min-Jung Kim, Hye-Jeong Yang, Soo-Cheol Choi, In-Ah Lee

**Affiliations:** 1 INVIVO Co., Ltd., Nonsan 32992, Republic of Korea; leeapf@nate.com (H.-Y.L.); pym07130@hanmail.net (Y.-M.P.); purity464@naver.com (H.-M.H.); sdy1325@hanmail.net (D.-Y.S.); hannav0117@hanmail.net (H.-N.J.); newstyle.kim@gmail.com (J.-G.K.); gydus0412@naver.com (H.-Y.P.); 2 Central Research and Development, Hanpoong Pharm & Foods, Wanju 55314, Republic of Korea; kimezz80@gmail.com (D.-S.K.); doublesky34@naver.com (J.-J.Y.); 3 Korea Food Research Institute, Wanju 55365, Republic of Korea; truka@kfri.re.kr (M.-S.K.); kmj@kfri.re.kr (M.-J.K.); yhj@kfri.re.kr (H.-J.Y.); 4 Department of Chemistry, Kunsan National University, Gunsan 54150, Republic of Korea

**Keywords:** osteoarthritis, *Kalopanax pictus* Nakai, *Achyranthes japonica* Nakai, anti-inflammation, MMPs

## Abstract

Osteoarthritis is a chronic inflammatory disease, and, due to the lack of fundamental treatment, the main objective is to alleviate pain and prevent cartilage damage. *Kalopanax pictus* Nakai and *Achyranthes japonica* Nakai are herbal plants known for their excellent anti-inflammatory properties. The objective of this study is to confirm the potential of a mixture extract of *Kalopanax pictus* Nakai and *Achyranthes japonica* Nakai as a functional raw material for improving osteoarthritis through anti-inflammatory effects in macrophages and MIA-induced arthritis experimental animals. In macrophages inflamed by lipopolysaccharide (LPS), treatment of *Kalopanax pictus* Nakai and *Achyranthes japonica* Nakai mixture inhibits NF-κB and mitogen-activated protein kinase (MAPK) activities, thereby inhibiting inflammatory cytokine tumor necrosis factor-alpha (TNF-α) and interleukin 6 (IL-6), inflammatory factors PGE2, MMP-2, and MMP-9, and nitric oxide (NO) was reduced. In addition, in an animal model of arthritis induced by MIA (monosodium iodoacetate), administration of *Kalopanax pictus* Nakai and *Achyranthes japonica* Nakai mixture reduced blood levels of inflammatory cytokines TNF-α and IL-6, inflammatory factors prostaglandin E2(PGE2), matrix metalloproteinase-2(MMP-2), and NO. Through these anti-inflammatory effects, MIA-induced pain reduction (recovery of clinical index, increase in weight bearing, and increase in area and width of the foot), recovery of meniscus damage, loss of cartilage tissue or inflammatory cells in tissue infiltration reduction, and recovery of the proteglycan layer were confirmed. Therefore, it is considered that *Kalopanax pictus* Nakai and *Achyranthes japonica* Nakai mixture has the potential as a functional raw material that promotes joint health.

## 1. Introduction

Osteoarthritis, the most common chronic inflammatory joint disease, leads to inflammation and pain due to the progressive degradation of joint cartilage and underlying bone tissue. This condition causes functional and mobility impairments, significantly reducing the quality of life [1,2,3,4,5,6]. One of the pathophysiological mechanisms of osteoarthritis involves chronic inflammation, which is characterized by immune cell infiltration in the synovium. As osteoarthritis progresses, the immune response initiated by T-cells leads to excessive proliferation and activation of synoviocytes, contributing to ongoing joint degeneration through the activation of cartilage-degrading enzymes [7,8,9].

The synovium, covered by a thin layer of synoviocytes, exhibits various observations, such as synoviocyte proliferation, angiogenesis, inflammatory cell infiltration, the expression of inflammatory cytokines, and protein-degrading enzymes in response to immune reactions [10]. Inflammatory synovium is often accompanied by significant hypoxia, and changes in oxygen tension within the joint due to joint movement result in hypoxia-reperfusion circulation. The metabolites and biochemical changes generated by these processes play a crucial role in the pathogenesis of most inflammatory diseases, directly causing oxidative damage to joint tissues and influencing the expression of various proteins involved in immune-inflammatory responses [11,12,13].

Factors associated with the onset of osteoarthritis can be broadly classified into B cells, T cells, and inflammatory cytokines. Proteolytic enzymes, cytokines, and nuclear transcription factor kB (NF-κB) are transcription factors of several genetic factors involved in immune and inflammatory responses. They promote the production of inflammatory cytokines, such as TNF-alpha, IL-6, and IL-17, and play a central role in the degeneration of cartilage tissue by damaging the collagen matrix that constitutes the cartilage tissue, particularly through the expression of enzymes, such as vascular endothelial growth factor (VEGF) and matrix metalloproteinases (MMPs) [14,15,16,17]. MMPs include collagenases (MMP-1, MMP-8, and MMP-13), stromelysins (MMP-3, MMP-7, MMP-10, and MMP-11), and gelatinases (MMP-2 and MMP-9), among which the gelatinase group rapidly increases its activity in an animal model of MIA-induced osteoarthritis, promoting matrix degradation of cartilage and subchondral bone [18,19,20].

Osteoarthritis is a chronic inflammatory disease characterized by pain, stiffness, and swelling. It is caused by various factors, including degenerative changes, immune system abnormalities, and trauma [21,22,23]. Since there is currently no definitive treatment to restore the affected joint in osteoarthritis, which is accompanied by chronic inflammation and symptoms such as pain, stiffness, and swelling, the primary objective of treatment is to alleviate pain and prevent cartilage damage. The ultimate goal is to control inflammation, reduce pain, delay or prevent joint damage, and maintain joint function to enhance the overall quality of life [24,25]. Common treatment methods include non-steroidal anti-inflammatory drugs (NSAIDs), painkillers, and intra-articular injections of hyaluronic acid. However, these treatments have drawbacks, such as potential side effects on the digestive, cardiovascular, renal, and coagulation systems, as well as concerns about long-term use. As a result, research on developing health-functional foods using natural ingredients with excellent safety is actively pursued [26,27,28].

*Kalopanax pictus* Nakai, belonging to the Araliaceae family, refers to the dried bark of *Kalopanax septemlobus* Koidz or the tree itself. It is known for its non-toxic nature, bitter and astringent taste, and significant antibacterial and antifungal properties. The main active components of *Kalopanax pictus* Nakai, including kalopanaxsaponins, liriodendrin, and syringin, have been reported to have analgesic and anti-inflammatory effects in animal models of rheumatoid arthritis [29,30,31].

*Achyranthes japonica* is a medicinal herb belonging to the Amaranthaceae family and is traditionally used in temperate and subtropical regions of China, Japan, Korea, and Southeast Asia [32,33]. It contains ecdysterones such as ecdysterone and inokosterone, alkaloids such as achyranthin, steroids like β-sitosterol, stigmasterol, rubrosterone, and various types of saponins. In oriental medicine, it is known for its effectiveness in inflammation, pain relief, muscle and bone strengthening, liver and kidney protection, improving blood circulation, treating skin diseases and boils, and alleviating joint pain [34,35].

In addition to *Kalopanax pictus* Nakai and *Achyranthes japonica* Nakai, several other herbs have been investigated for their potential effects on inflammation and osteoarthritis. For example, studies have shown that *Curcuma longa* (turmeric) possesses anti-inflammatory properties and can alleviate symptoms of osteoarthritis [36]. Another herb, Boswellia serrata, has been reported to exhibit anti-inflammatory effects and may have a beneficial impact on osteoarthritis [37,38]. Furthermore, *Zingiber officinale* (ginger) has shown anti-inflammatory activity and may contribute to the management of osteoarthritis symptoms [39]. These studies highlight the potential of various herbs in modulating inflammation and alleviating osteoarthritis symptoms. However, the specific effects of these herbs on inflammation and osteoarthritis require further investigation.

In this study, in pathological management, pharmacotherapy aims to achieve pain relief, inflammation suppression, and analgesic effects and we investigated the effects of a combination of *Kalopanax pictus* Nakai and *Achyranthes japonica* Nakai complex extract on osteoarthritis using a monosodium iodoacetate (MIA)-induced animal model. Despite extensive research having been conducted on the effects of *Kalopanax pictus* and *Achyranthes japonica* extracts in experimental mouse models of osteoarthritis, this study was designed to investigate the synergistic efficacy and derive superior effects. While previous studies have examined the individual use of *Kalopanax pictus* Nakai and *Achyranthes japonica* Nakai mixture extracts, there have been no studies on their combined use in specific ratios. We confirmed the anti-inflammatory and joint-improving effects of the extract and evaluated the potential of *Kalopanax pictus* Nakai and *Achyranthes japonica* Nakai complex extract as a functional food ingredient for joint and bone health.

## 2. Materials and Methods

### 2.1. Experimental Material

We extracted *Kalopanax pictus* Nakai (50 g) and *Achyranthes japonica* Nakai (100 g) twice with 10-fold weight of 50% (*v*/*v*) ethanol at 80–85 °C for 3 h, and then filtered through a 5-μm filter. 25.6 g of the mixed powder obtained by evaporating the filtrate and vacuum drying was named SHP-47B. In subsequent experiments, dry powder SHP-47B was used for experiments.

### 2.2. The Quantitative HPLC Analysis

The quantitative HPLC (high-performance liquid chromatography) analysis methods for SHP-47B were designed complying with liquid chromatography of General Tests in the Korean Pharmacopoeia Twelfth Edition [40]. All quantitative HPLC analyses were performed in triplicate.

The quantitative analysis for the marker compound liriodendrin of *Kalopanax pictus* Nakai were performed with HPLC (LC-2030 C 3D; Shimadzu Corporation, Kyoto, Japan) equipped with Atlantis T3 column (4.6 mm ID × 250 mm L, 5 μm particle size; Waters, Milford, MA, USA). The mobile phases were consisted of A (0.1% phosphoric acid in DW) and B (acetonitrile). The gradient system was composed as follows: 4% B (0 min), 5% B (7 min), 20% B (25 min), 20% B (40 min), 30% B (50 min), 4% B (52 min), 4% B (60 min). The flow rate was 1 mL/min with a detection wavelength of 210 nm [41,42]. The standard solution of liriodendrin was prepared at a concentration of 40 μg/mL in the 50% MeOH. The test solution for the quantitative analysis of liriodendrin was prepared by sonication of 0.2 g of SHP-47B in 50 mL of 50% MeOH during 60 min followed by filtration using 0.45 um syringe filter. The injection volume was 20 μL each of the test solution and the standard solution.

The marker compound, ecdysterone, of *Achyranthes japonica* was quantified using HPLC (LC-2030; Shimadzu Corporation, Kyoto, Japan) with a Capcell Pak C18 column (4.6 mm ID × 250 mm L, 5 μm particle size; Osaka Soda Co. Ltd., Osaka, Japan). The HPLC analyses were conducted at a flow rate of 1 mL/min, a column temperature of 35 ℃, and a detection wavelength of 254 nm. The gradient conditions of the mobile phase were isocratic 15% MeCN from 0 min to 8 min, gradient 15–30% MeCN from 8 min to 15 min, isocratic 30% MeCN from 15 min to 30 min, gradient 30–15% MeCN from 30 min to 31 min, and isocratic 15% MeCN from 31 min to 40 min. The standard solution of ecdysterone was processed at a concentration of 50 μg/mL in 50% MeOH. The ecdysterone-test solution was prepared similarly to those of liriodendrin-test solution except for duration of sonication, which was performed for 90 min.

### 2.3. Writhing Test

For the writhing test, a mouse was administered interaperioneally with 0.5 mL of 1% acetic acid dissolved in saline. Immediately after the injection, the animals were placed in an acryl observation changer (20 cm high, 20 com diameter). The number of writhes was counted during a 30 min period following the injection of acetic acid. A writhe was defined as a contraction of the abdominal muscles accompanied by an extension of the forelimbs and elongation of the body. The number of animals used for each group was five.

### 2.4. Cell Viability and NO Production in RAW 264.7 Cells

RAW264.7 cells, a murine macrophage cell line, were purchased from the Korea Cell Line Bank (KCLB40071) and used. Reagents required for cell culture were purchased from Gibco (Houston, TX, USA), and the medium was Dulbecco’s Modified Eagle Medium (DMEM) with 10% fetal bovine serum (Ryun) and 1% antibiotics-antimycotic added to the cells at 37 °C in the presence of 5% CO_2_. It was used in the experiment while being subcultured once every 2–3 days in an incubator.

For analysis of cell viability, 2 × 10^4^ cells/90 μL/well were treated with samples for each concentration and incubated at 37 °C and 5% CO_2_ for 24 h each. After that, 10 μL of WST-1 (ITSBio, Seoul, the Republic of Korea) solution was added to 100 μL of the cell culture medium, incubated for 1 h, and the absorbance value was measured using a Multi Detection Reader (Infinite 200, TECAN Group Ltd., Männedorf, Switzerland). The control group was set as an experimental group treated with the same concentration as the high-concentration experimental group, only with the solvent dissolved in the sample without processing the sample. Cell viability was calculated according to the following formula.

cell viability (%) = (sample treatment group/control group) × 100

For NO measurement, Raw 264.7 cells were dispensed in a 24-well plate to be 8 × 10^4^ cells/well and cultured for 24 h. After that, the samples were treated and reacted by concentration, and each well was treated with LPS and cultured for 24 h. After incubation, 100 μL of the culture supernatant was taken, the same amount of Griess reagent was added, it was left it for 10 min, and the absorbance at 540 nm was measured. The concentration of nitrite produced was calculated using a standard curve in which sodium nitrite was dissolved in DMEM medium, and the NO production inhibitory activity of each sample was confirmed based on the difference in the amount of nitrite produced.

### 2.5. Inflammation-Related Cytokine Production Analysis in RAW264.7

For inflammatory cytokine measurement, Raw 264.7 cells were dispensed in a 24-well plate to be 8 × 10^4^ cells/well and cultured for 24 h. After that, the samples were treated and reacted by concentration, and each well was treated with LPS and cultured for 24 h. After incubation, the supernatant of the cell lysate was centrifuged. The inflammatory cytokines TNF-α (CSB-E11987r, Cusabio, TX, USA), IL-6 (CSB-E04640r, Cusabio, TX, USA), PGE2 (CSB-E07967r, Cusabio, TX, USA), MMP-2 (ab213910, Abcam, Cambridge, UK), and MMP-9 (RMP900, R&D Systems, Minneapolis, MN, USA) were measured using ELISA kits, following the manufacturers’ instructions. The experimental results of inflammatory cytokines are shown in Figure S1.

### 2.6. Western Blot Analysis

After dispensing the cells at 2 × 10^6^ cells/mL in a 100 π dish, the sample was treated and cultured for 24 h. After that, it was washed three times with cold PBS and protein was extracted from the cells using lysis buffer (PRO-PREPTM protein extraction solution, InTron, Korea). was extracted. The extracted protein was quantified with Bradford reagent (Bio-Rad, HerculesL, CA, USA) and the same amount was used for Western blot. For Western blot, the same amount of protein was electrophoresed on SDS-PAGE gel, transferred to PVDF membrane, and blocked with 5% skim milk solution for 1 h. After that, the primary antibody against the target protein was added overnight at 4 °C, and the secondary antibody containing HRP was treated at room temperature for 1 h. Then, it was analyzed by scanner (LI-COR, Lincoln, NE, USA). TBS-Tween 20 solution was used for each step of washing, and the antibodies used in the experiment are shown in Table S1.

### 2.7. Animals and Experimental Design

As the experimental animal, an eight-week-old male Sprague-Dawley (SD) rat in a specific-pathogen-free (SPF) state was purchased from Samtaco Bio Korea (Osan, Korea), and was acclimatized for seven days and used in the experiment. During the breeding period, regular solid feed (Samtako, Gyunggi, Korea) was consumed, and, during the acclimatization period, filtered drinking water was changed daily and freely consumed. During the animal breeding period, the temperature was 23 ± 1 ℃, the humidity was 50 ± 5%, the noise was less than 60 phone, the lighting time was 08:00~20:00 (12 h a day), the illuminance was 150~300 Lux, the ventilation was 10 times per hour, and 12 environments were maintained. The use of mice was reviewed and approved by Invivo Animal Care Committee (IACUC approval number: IV-RB-02-2204-09).

Once the acclimatization period was over, the experimental animals were separated using the egg mass method based on body weight so that the average value between groups was uniform. The experimental group was a normal group that did not induce arthritis, a control group that did not treat the sample after inducing arthritis (control), and a treatment group by SHP-47B concentration (50 mg/kg, 100 mg/kg, and 300 mg/kg) after induction of arthritis, and as a positive control group, Celecoxib was administered (30 mg/kg), and 10 heads per group were set. Samples were orally administered for a total of six weeks, and the control group was administered the same amount of distilled water as the experimental group.

The establishment of an arthritic animal model was carried out by injecting 50 μL (3 mg/mL) of MIA (monosodium iodoacetate) into the joint cavity of the left knee with an insulin syringe, followed by sample administration for three weeks before MIA injection (before arthritis induction) and after MIA injection (after induction of arthritis). The arthritis improvement effect was demonstrated by sample administration for three weeks. MIA was used diluted with 0.9% sodium chloride [43,44].

### 2.8. Weight-Bearing Index and Arthritis Clinical Index Analysis

The arthritis clinical index was independently observed by four experimenters, and the swelling and the bending of the knee joint in each experimental group were scored as no change (0 point) and 1–3 points, depending on the degree of severity. Scores were scored and expressed as an average value.

The hindlimb weight bearing was set up at a 60 degree inclination in a plastic room using an incapacitance meter tester (IMT), and, then, the strength applied to each hindlimb was averaged over 10 s. The percentage of body weight distributed over the treated ipsilateral hind limb was calculated using the following equation:

weight-bearing index (%) = (weight of evoked lower extremity/weight of normal lower extremity) × 100

### 2.9. Gait Analysis

In the case of gait analysis (paw area and paw width), ink was applied to the hind paw and measured by running the experimental animal on a white paper with a length of 60 cm and a width of 7 cm, and footprints were measured using Image J software version 2023.

### 2.10. Serum Biochemical Analysis

Blood was collected from the abdominal vena cava after inhalational anesthesia, and divided into EDTA tubes and conical tubes for analysis. For hematological examination, blood was put into an EDTA tube (DB Caribe, Ltd., Washington, DC, USA), it was rotated on a roll mixer for about 30 min, and, then, a blood analyzer (Hemavet 950Fs, Drew Scientific Inc., Dallas, TX, USA) was used to measure white blood cells, lymphocytes, granulocytes, and mid-size cells.

On the other hand, the blood collected in the conical tube was coagulated at room temperature for 30 min for cytokine analysis, and the serum separated in a centrifuge at 3000 rpm for 10 min was used with an ELISA kit to detect the inflammatory cytokine TNF-α (CSB-E11987r, Cusabio, TX, USA), IL-6 (CSB-E04640r, Cusabio, TX, USA), PGE2 (CSB-E07967r, Cusabio, TX, USA), MMP-2 (ab213910, Abcam, Cambridge, UK), and nitric oxide (ab65328, Abcam, Cambridge, UK) contents were measured.

### 2.11. Micro-CT Analysis

Micro-CT measurement was taken for 3 min using a Quantum FX Micro-CT (Perkin Elmer, Waltham, MA, USA) set at a tube voltage of 90 kVp, tube current of 160 μA, and FOV of 10 mm, and image analysis was performed using Analyze 12 (Mayo Clinic, Scottsdale, AZ, USA). In this analysis, volume rendering was performed after the experiment was completed to separate the meniscus, and then a 3D image was obtained.

### 2.12. Histological Analysis

The excised tissue was fixed in 10% formalin solution after cutting (trimming) the specimen fixed in 10% formalin solution. Then, each tissue was embedded in paraffin, sectioned to a thickness of 3 μm, cut, stained with hematoxylin–eosin or safranin-o, and observed under an optical microscope. The staining results of all tissues were read by pathology experts using an optical microscope, and were read blindly so that the experimental group could not be known in advance to exclude subjective judgment.

### 2.13. Statistical Analysis

All experimental results were calculated as mean ± standard error (mean ± S.E.) using a statistical program (SPSS ver. 12.0, SPSS Inc., Chicago, IL, USA). Statistical analysis according to the statistical significance test between each experimental group was performed with ANOVA (one-way analysis of variance test), and, if there was significance, post-testing was performed with Duncan’s multiple range test when *p* < 0.05 was less.

## 3. Results

### 3.1. The Quantitative HPLC Analysis

**Figure 1:**
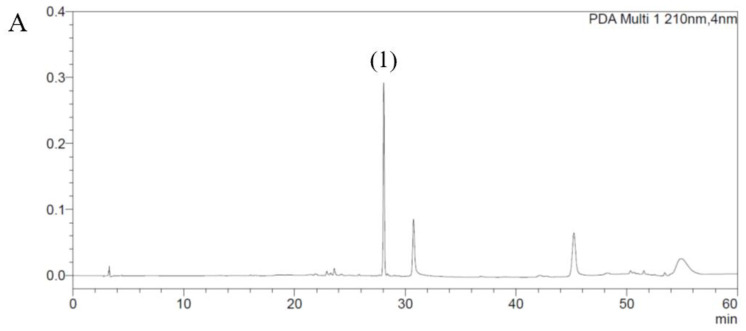

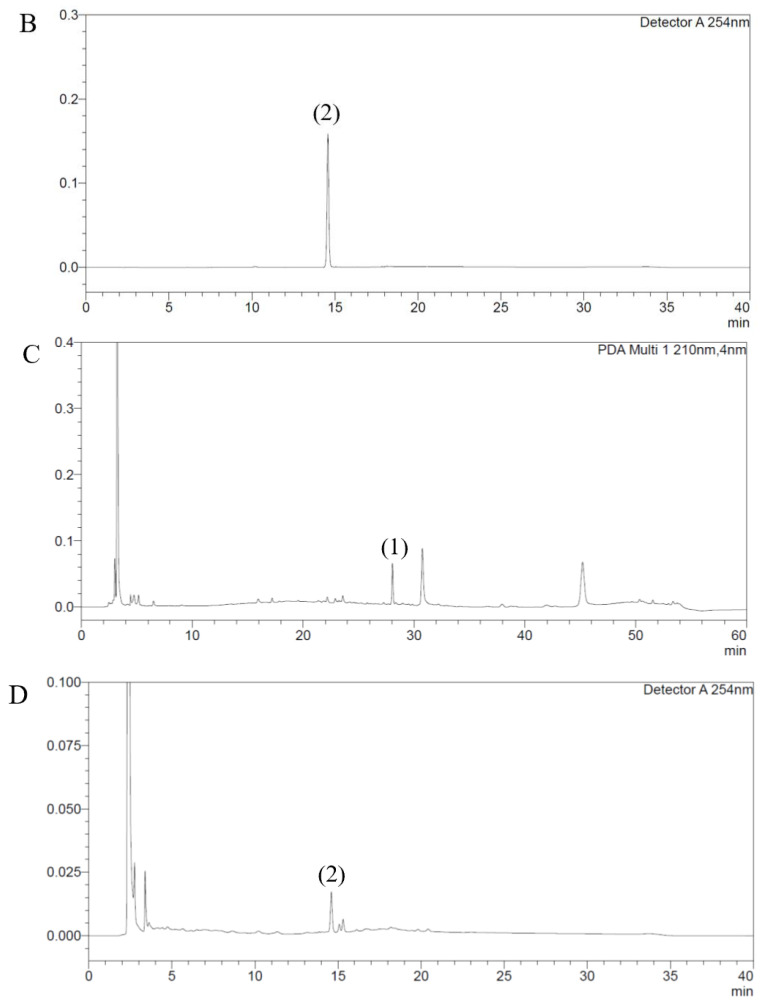
Chromatograms of liriodendrin-standard solution (**A**), ecdysterone-standard solution (**B**), liriodendrin-test solution (**C**), and ecdysterone-test solution (**D**). (**1**): Liriodendrin and (**2**): Ecdysterone.

The contents of the marker compounds for SHP-47B were determined by the quantitative HPLC analysis. The results indicated that the amount of liriodendrin and ecdysterone in SHP-47B were 1.74 ± 0.05 mg/g and 1.18 ± 0.05 mg/g, respectively (Figure 1).

### 3.2. Writhing Test

The writhing test was divided into first and second, and, finally, the compounds to be used in the experiment were identified. In the first test, SHP-43 and SHP-47 were judged to be the most suitable compounds for the pain test, and, among them, SHP-47 (*Achyranthes japonica*) was judged to be the most suitable. In the second test, *Achyranthes japonica* and *Kalopanax pictus* Nakai were mixed in various ratios to make SHP-47A~D mixtures and a writhing test was conducted (Table 1).

As a result, it was judged that SHP-47B was most suitable for the pain and active part, and SHP-47B was selected and used in subsequent experiments.

### 3.3. Cell Viability and NO Production in RAW 264.7 Cells

**Figure 2:**
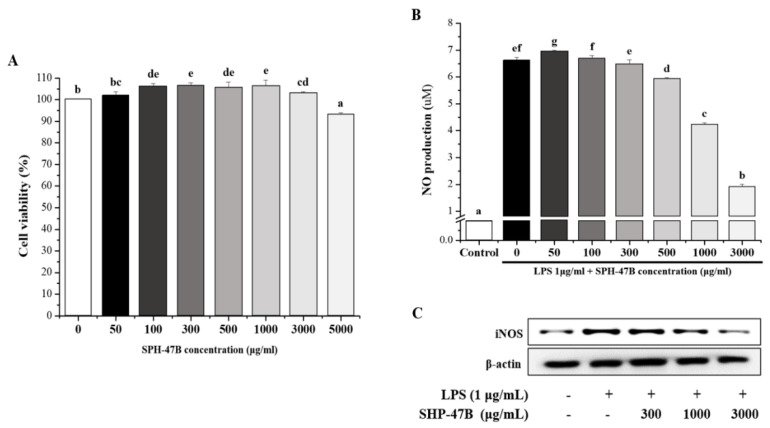
In RAW 264.7 cells, the (**A**) cell viability of SHP-47B and the physiological activity of (**B**) NO and (**C**) iNOS were measured. Cell viability was measured by WST-1 assay, and after measuring NO production, iNOS was measured by Western blot analysis. The data are expressed as the mean ± SD (*n* = 3), and different letters (g > f > e > d > c > b > a) indicate a significant difference at *p* < 0.05, as determined by Duncan’s multiple-range test.

The cell viability of SHP-47B at various concentrations (0 to 5000 μg/mL) is presented in Figure 2A. As a result of measuring cell viability after treating RAW 264.7 cells with SHP-47B for 24 h, toxicity to cells was not confirmed from SHP-47B 50 μg/mL to 3000 μg/mL concentration, but significant at 5000 μg/mL concentration reduction was confirmed. Based on these results, subsequent experiments were conducted by setting the highest concentration to 3000 μg/mL.

Referring to the cell results, the expression of NO product and iNOS protein was confirmed by treating SHP-47B at each concentration in RAW 264.7 inflamed with LPS. NO production significantly increased to 6.63 ± 0.11 μM in the LPS-treated group, and NO production was decreased in a concentration-dependent manner in the SHP-47B-treated group. In particular, it was confirmed that NO production was reduced to 1.92 ± 0.09 μM in the 3000 μg/mL treatment group (Figure 2B).

Additionally, as a result of measuring iNOS protein expression in Western blot results, it was confirmed that iNOS protein expression was suppressed in a concentration-dependent manner in the SHP-47B treated group (Figure 2C).

### 3.4. Western Blot Analysis

**Figure 3:**
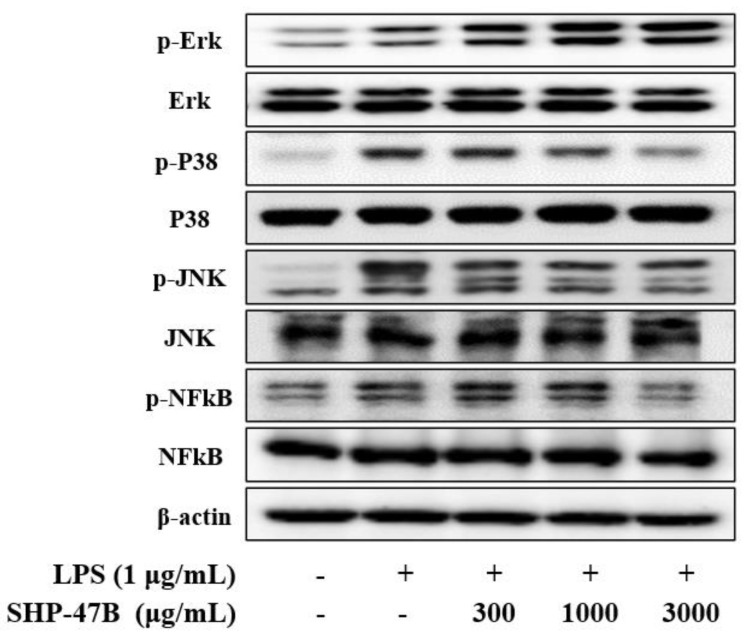
Analysis of the physiological activity of SHP-47B on the activation of NF-κB and MPAK pathways in LPS-induced inflammation in RAW 264.7 macrophages. Proteins obtained from RAW 264.7 cells were analyzed by Western blot for the expression of proteins related to each of the NF-κB and MAPK pathways.

NF-κB and MAPK signaling pathways are activated during inflammatory processes and promote the expression of inflammatory mediators. Based on this mechanism, the effects of SHP-47B on the NF-κB and MAPK signaling pathways in LPS-induced RAW 264.7 cells were confirmed through Western blot results. As demonstrated in Figure 3, NF-κB and MAPK-related protein expression levels increased in the LPS-only treatment group, and protein expression levels were suppressed in a concentration-dependent manner except for p-ERK in the SHP-47B treatment group.

### 3.5. Weight-Bearing Index and Arthritis Clinical Index Analysis

**Figure 4:**
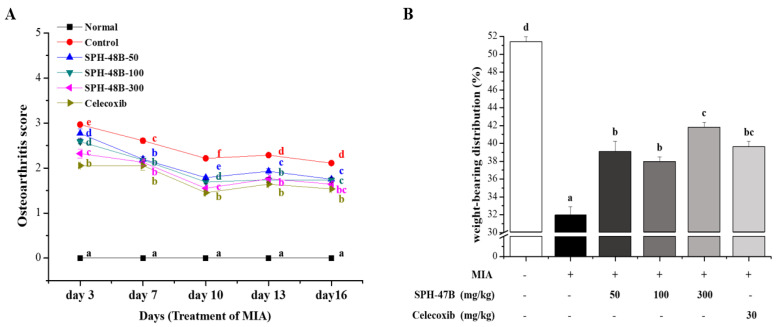
Effects of SHP-47B treatment on (**A**) weight bearing and (**B**) clinical arthritis in MIA-induced arthritis mice model. The data are expressed as the mean ± SD (*n* = 7), and different letters (f > e > d > c > b > a) indicate a significant difference at *p* < 0.05, as determined by Duncan’s multiple-range test.

To investigate the efficacy of SHP-47B in a mice model of arthritis induced by MIA, the weight-bearing index and the clinical index of arthritis were measured. A total of five measurements were taken from the induction of arthritis (MIA injection) to the end of the experiment. From the first three days after the onset of arthritis, the normal group showed a normal gait, but the control group and the experimental group showed pain and leg dragging during walking due to arthritis. As a result expressed as a clinical index, the control group showed 2.96 ± 0.04, the SHP-47B treatment group showed 2.77 ± 0.11 in the 50 mg/kg group, 2.59 ± 0.05 in the 100 mg/kg group, and 2.32 ± 0.10 in the 300 mg/kg group. The celecoxib group used as a positive control showed a result of 2.05 ± 0.04 (Figure 4A).

Once the experiment was completed, the weight-bearing index was analyzed to confirm the pain amelioration effect of SHP-47B in an animal model of arthritis induced by MIA. From the analysis results, it was confirmed that the result value of 31.95 ± 0.95% of the control group was significantly reduced compared to the result of 51.39 ± 0.57% of the normal group. In contrast, in the case of the SHP-47B treatment group, the result values were 39.08 ± 0.17% in the 50 mg/kg group, 37.89 ± 0.60 in the 100 mg/kg group, and 41.78 ± 0.61% in the 300 mg/kg group, and the positive control group (celecoxib) used as a weight-bearing index result value of 39.62 ± 0.59% (Figure 4B).

### 3.6. Gait Analysis

**Figure 5:**
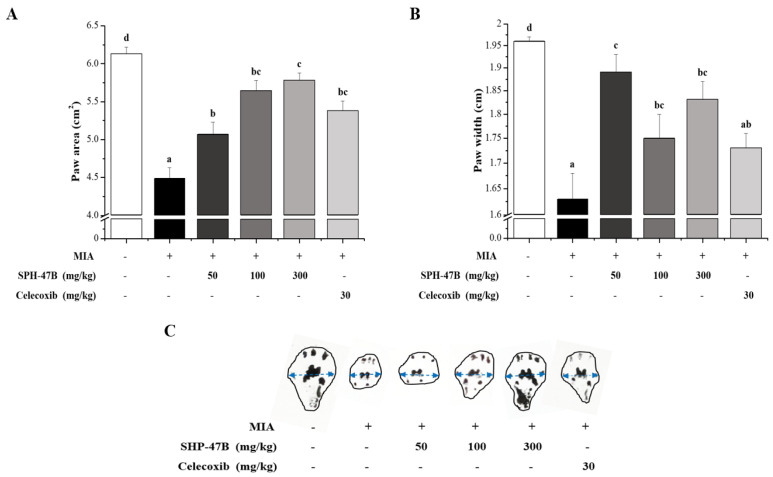
The effects of SHP-47B treatment on plantar (**A**) area and (**B**) width in gait analysis of an mice model of arthritis induced by MIA. Gait analysis results were comprehensively expressed in the (**C**) mouse plantar measurement picture. The data are expressed as the mean ± SD (*n* = 7), and different letters (d > c > b > a) indicate a significant difference at *p* < 0.05, as determined by Duncan’s multiple-range test.

As another method to confirm the pain improvement effect of SHP-47B in mice model of arthritis induced by MIA, the foot area and width were measured and analyzed in the animal model walking. First, as a result of measuring the foot area, it was confirmed that the normal group was 6.13 ± 0.09 cm^2^, while the control group was 4.49 ± 0.14 cm^2^. On the other hand, for the SHP-47B treated group, it was 5.07 ± 0.16 cm^2^ in the 50 mg/kg group, 5.64 ± 0.14 cm^2^ in the 100 mg/kg group, and 5.78 ± 0.10 cm^2^ in the 300 mg/kg group. These results confirmed that the pain caused by arthritis was suppressed in a concentration-dependent manner in the SHP-47B treatment group, resulting in an increase in the area and width of the foot during walking. In particular, in the 100 mg/kg and 300 mg/kg groups, it was confirmed that the area and width of the foot increased compared to the 5.37 ± 0.14 cm^2^ result of the celecoxib group used as a positive control group (Figure 5A).

As a result of foot width analysis, it was confirmed that the result value of 1.96 ± 0.01 cm in the normal group was significantly reduced compared to the result of 1.63 ± 0.05 cm in the control group. On the other hand, in the case of the SHP-47B treatment group, the width increased to 1.89 ± 0.04 cm in the 50 mg/kg group, 1.75 ± 0.05 cm in the 100 mg/kg group, and 1.83 ± 0.04 cm in the 300 mg/kg group. The celecoxib group used as a positive control showed a result of 1.73 ± 0.03 cm. The foot width increased in all experimental groups compared to the control group, and it was confirmed that the foot width increased more than the Celecoxib treated group at all concentrations in the SHP-47B treated group (Figure 5B).

Through these results, it was shown that the treatment of SHP-47B suppressed the pain caused by arthritis, resulting in an increase in the area and width of the foot during walking.

### 3.7. Serum Biochemical Analysis

For the results reported in Table 2, hematological analysis was performed using blood immune cells of animal models in MIA-induced arthritis experiments. As a result of hematological analysis, the analysis of the white blood cell (WBC) count measurement result and the change in blood mid-cells (MID) content could not confirm a significant change between each experimental group. However, significant changes were observed between each experimental group in the content of granulocytes (GRA) in the blood and the change in the number of lymphocytes (LYM) in the blood.

As a result of analyzing the content of granulocytes (GRA) in the blood, the control group had 1.28 ± 0.05 × 10^3^/μL (15.21 ± 0.50%) compared to 2.03 ± 0.18 × 10^3^/μL (22.63 ± 1.39%) of the normal group, indicating the induction of arthritis by MIA. Through this, it was confirmed that the content of granulocytes in the blood was significantly reduced. On the other hand, in the case of the SHP-47B treatment group, 1.51 ± 0.11 × 10^3^/μL (17.24 ± 1.33%) in the 50 mg/kg group, 1.61 ± 0.12 × 10^3^/μL (16.61 ± 0.71%) in the 100 mg/kg group, and 1.41 ± 0.10 × 10^3^/μL (19.37 ± 0.94%) in the 300 mg/kg group. In the celecoxib group used as a positive control group, 1.67 ± 0.05 × 10^3^/μL (20.93 ± 0.84%) appeared, confirming a significant increase compared to the control group in the 300 mg/kg and celecoxib groups.

As a result of measuring the change in blood lymphocytes (LYM), the control group was 6.20 ± 0.30 × 10^3^/μL (80.10 ± 0.95%) compared to 6.21 ± 0.51 ×10^3^/μL (70.67 ± 1.52%) of the normal group, indicating that arthritis was induced by MIA. Through this, it was confirmed that the content of lymphocytes in the blood was increased. On the other hand, in the case of the SHP-47B treatment group, 6.33 ± 0.32 ×10^3^/μL (75.40 ± 1.03%) in the 50 mg/kg group, 7.08 ± 0.50 × 10^3^/μL (77.69 ± 1.01%) in the 100 mg/kg group, and 5.14 ± 0.19 × 10^3^/μL (74.96 ± 0.96%) in the 300 mg/kg group. In the celecoxib group used as a positive control group, it was 5.74 ± 0.27 × 10^3^/μL (73.61 ± 0.92%). Through these results, a significant decrease was confirmed in the SHP-47B and celecoxib groups compared to the control group.

**Figure 6:**
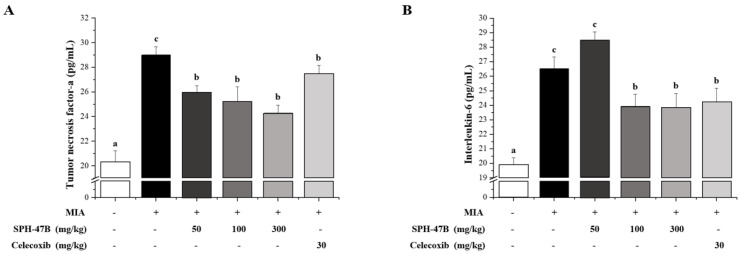

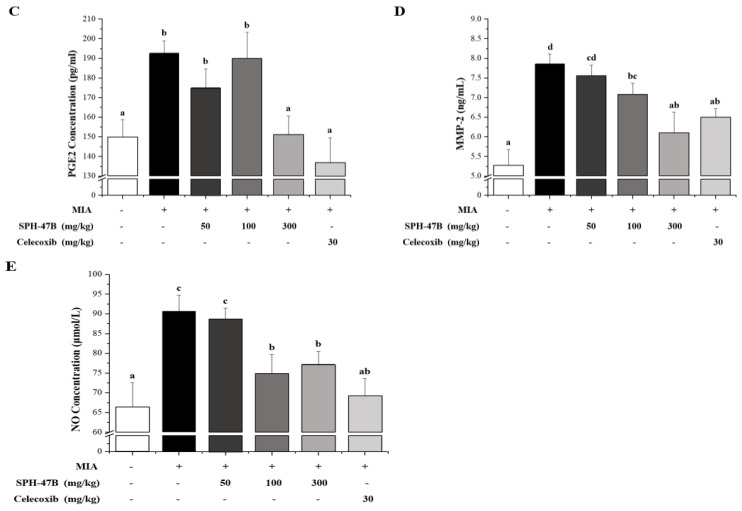
Effect of SHP-47B treatment on hematological analysis, (**A**) tumor necrosis factor-α (TNF-α), (**B**) interleukin-6 (IL-6), (**C**) prostaglandin E2 (PGE2), (**D**) matrix metalloproteinase-2 (MMP-2), and (**E**) nitric oxide (NO) in a mice model of arthritis induced by MIA. The data are expressed as the mean ± SD (*n* = 7), and different letters (d > c > b > a) indicate a significant difference at *p* < 0.05, as determined by Duncan’s multiple-range test.

As a result of blood inflammatory cytokine analysis using an ELISA kit, it was confirmed that the expression levels of TNF-α, IL-6, PGE2, MMP-2, and NO were significantly increased in the serum of mice with arthritis induced by MIA. In the TNF-α result, the SHP-47B treatment group inhibited TNF-α in a concentration-dependent manner, and showed a stronger inhibitory effect than celecoxib used as a positive control group (Figure 6A). In the IL-6 results, the SHP-47B 100 mg/kg and 300 mg/kg groups effectively inhibited IL-6, and showed similar inhibitory effects to celecoxib used as a positive control group (Figure 6B) In the PEG2 results, the SHP-47B 300 mg/kg group showed a significant inhibitory effect, and showed a similar level of inhibitory effect to the normal group (Figure 6C). In the results of MMP-2, the SHP-47B treatment group inhibited MMP-2 in a concentration-dependent manner, and the 300 mg/kg group showed a stronger inhibitory effect than celecoxib used as a positive control group (Figure 6D). Finally, in the NO results, the SHP-47B treatment group suppressed NO in a concentration-dependent manner, and the 100 mg/kg and 300 mg/kg groups showed similar inhibitory effects to celecoxib used as a positive control (Figure 6E).

### 3.8. Micro-CT Analysis

**Figure 7:**
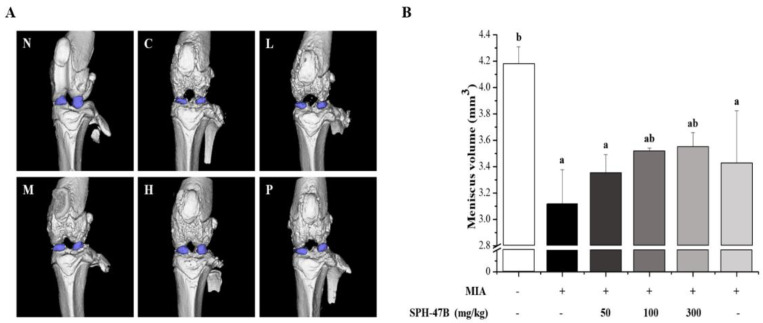
Effects of SHP-47B treatment on (**A**) meniscus volume and (**B**) micro-CT images (blue-purple: meniscus) in an MIA-induced arthritis mice model. N: normal, C: control, L: SHP-47B 50 mg/kg, M: SHP-47B 100 mg/kg, H: SHP-47B 300 mg/kg, P: celecoxib. The data are expressed as the mean ± SD (*n* = 3), and different letters (b > a) indicate a significant difference at *p* < 0.05, as determined by Duncan’s multiple-range test.

The effect of SHP-47B on knee joint meniscus volume was analyzed in an animal model of MIA-induced arthritis using micro-CT-arthrography (Figure 7A). The meniscus is a half-moon-shaped piece of cartilage located between the upper and lower joints of the knee. Located on the inside and outside of the knee, it is known as a tissue that protects the joint, absorbs shock, and helps the knee to function properly.

As a result of Micro CT analysis, the meniscal volume of the control group was 3.12 ± 0.26 mm^3^ compared to 4.18 ± 0.13 mm^3^ of the normal group, confirming that the meniscal volume was significantly reduced through arthritis caused by MIA. On the other hand, in the case of the SHP-47B treatment group, meniscus volume increased by 3.35 ± 0.14 mm^3^ in the 50 mg/kg group, 3.52 ± 0.02 mm^3^ in the 100 mg/kg group, and 3.55 ± 0.11 mm^3^ in the 300 mg/kg group. The celecoxib group used as a positive control showed a result of 3.43 ± 0.40 mm^3^. Through these results, SHP-47B treatment increased the meniscal volume of the knee joint and showed efficacy against arthritis (Figure 7B).

### 3.9. Histological Analysis

**Figure 8:**
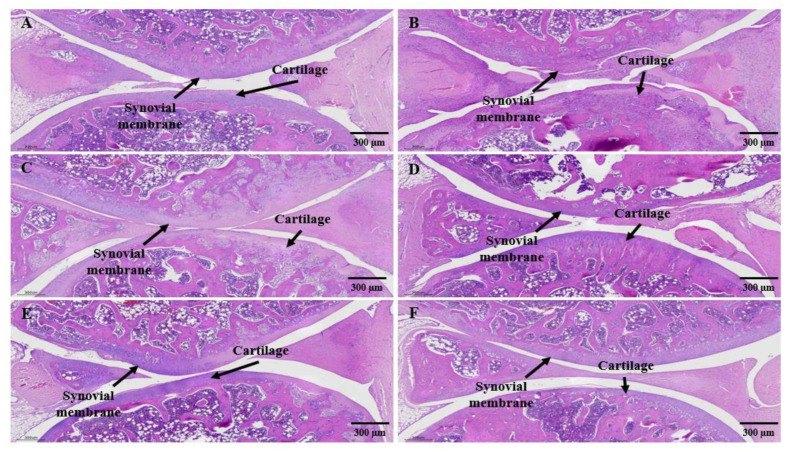
Analysis of synovial cell changes and inflammatory cell infiltration in the knee joint by H&E staining in an MIA-induced arthritis mouse model. (**A**) normal group, (**B**) control group, (**C**) SHP-47B 50 mg/kg group, (**D**) SHP-47B 100 mg/kg group, (**E**) SHP-47B 300 mg/kg group, and (**F**) positive control (celecoxib) group. Histological analysis result image magnification = 4×, and scale bar = 300 μm.

**Figure 9:**
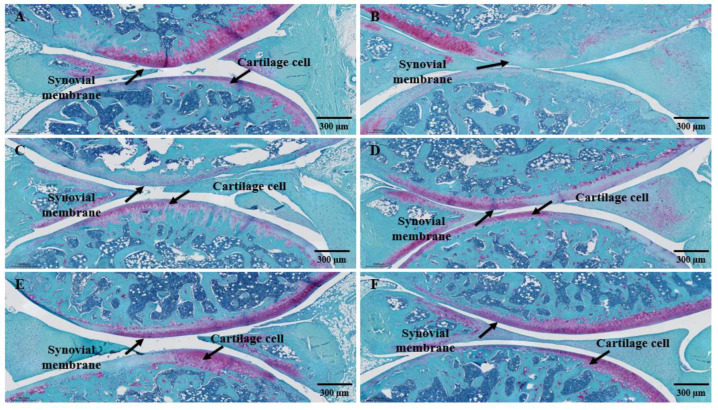
Analysis of cartilage tissue damage through Safranin-O staining in MIA-induced arthritis mouse model. (**A**) normal group, (**B**) control group, (**C**) SHP-47B 50 mg/kg group, (**D**) SHP-47B 100 mg/kg group, (**E**) SHP-47B 300 mg/kg group, and (**F**) positive control (celecoxib) group. Histological analysis result image magnification = 4×, and scale bar = 300 μm.

For histopathological analysis using hematoxylin and eosin (H&E) and Safranin-O fast green staining, the left knee joint of the MIA-induced arthritis mouse model was excised and the articular cartilage was analyzed using Motic EasyScan (Motic, Xiamen, China) (Figure 8 and Figure 9).

As a result of analyzing changes in synovial cells and infiltration of inflammatory cells in the knee joint by H&E staining, synovial tissues were regularly arranged and no inflammatory cells were found in the joints of the normal group (Figure 8A). On the other hand, in the control group, severe bone erosion of cartilage tissue around the joint and infiltration of inflammatory cells in the tissue were markedly observed, and synovial inflammation thickening was also observed (Figure 8B). In the SHP-47B test group, as the administration concentration increased, the loss of cartilage tissue and the infiltration of inflammatory cells into the tissue gradually decreased (Figure 8C,D). Especially, in the 300 mg/kg group, no inflammatory cell infiltration was observed in the tissue, and the tissue arrangement was relatively uniformly arranged (Figure 8E,F).

As a result of checking the cartilage tissue damage through Safranin-O staining of the proteoglycan layer of cartilage cells in the knee cartilage (Figure 9), in the normal group, the cartilage cells of the contact, transition, and radial areas of the articular cartilage were arranged in parallel, and the cartilage matrix was also was well kept (Figure 9A). However, in the control group, there was loss of proteoglycan due to inflammatory findings and cartilage invasion, and extensive destruction of subchondral bone tissue was observed (Figure 9B). In the SHP-47B test group, as the administration concentration increased, the cartilage layer was clearly observed (Figure 9C,D), and, in the 300 mg/kg group, the improvement of the cartilage layer was observed at a level similar to that of the normal group together with celecoxib, a positive control group (Figure 9E,F).

The staining results of all tissues were read by pathology experts using an optical microscope, and were read blindly so that the experimental group could not be known in advance to exclude subjective judgment.

## 4. Discussion

Osteoarthritis is a representative inflammatory joint disease that causes pain, stiffness, and swelling due to degenerative changes, immune system abnormalities, trauma, and other factors, leading to functional and mobility impairments in daily life and reducing quality of life [1,2,3,19]. The treatment goal for patients with arthritis is to reduce pain through inflammation control and to minimize joint deformation and disability by preventing joint damage, thereby improving the quality of life by maintaining joint function as much as possible [22,23].

The main active compounds in *Kalopanax pictus* Nakai used in the research are kalopanaxsaponins, liriodendrin, and syringin, which have been reported to possess analgesic and anti-inflammatory effects in a rheumatoid arthritis animal model [27,28,29]. HPLC analysis revealed that liriodendrin had a higher content compared to other plant compounds and was recommended as a marker compound [20]. Additionally, ecdysterone, known for their efficacy in promoting the growth and activity of osteoblasts and osteoclasts, were combined with liriodendrin to create the SHP-47B compound and investigated the possibility of SHP-47B as a functional ingredient for improving joint health [45,46,47].

The immunological causes of osteoarthritis are known to involve various cytokines such as B cells, T cells, TNF-α, IL-1, IL-6, and IL-17, among others. Osteoarthritis is known to be promoted by inflammatory cytokines (TNF-α, IL-1, IL-6, IL-17, etc.) that stimulate the secretion of matrix metalloproteinases (MMPs) from synovial fibroblasts [48,49,50,51,52,53]. In addition, it can be said that the homeostasis of pro-inflammatory and anti-inflammatory cytokines is disrupted through the activation of the diverse and complex cytokine network of rheumatoid arthritis, leading to a state where inflammation is exacerbated. Proteolytic enzymes, such as matrix metalloproteinase (MMPs), play an important role in cartilage and bone destruction in inflammatory osteoarthritis. MMPs act as extracellular matrix proteins in vivo and are involved in proteoglycan degradation in osteoarthritis, and regulation of MMP activity is important in arthritis treatment [54,55].

Currently, through research on these activation mechanisms, kinases, such as JAK, MAPK, SYK, PI3K, NF-κB, and BTK, are being recognized as major targets for the development of rheumatoid arthritis treatments. In particular, the JAK-STAT pathway is reported to be a very important target for the development of rheumatoid arthritis therapies [56,57].

In this study, the anti-inflammatory mechanism of thawed hull extract was identified in vitro and in vivo, and its potential as a functional material for improving osteoarthritis was confirmed. First, in RAW264.7, in which inflammation was induced by LPS, treatment with thaw extract inhibited the activity of MAPK and NF-κB, which are signal transduction pathways activated by Toll-like receptor (TLR). These results confirmed that strong inhibition of inflammatory cytokine (TNF-α, IL-1, IL-6) and inflammatory mediators (NO, PGE2) [58,59,60].

NO is known to play a role in inhibiting tumor growth by mediating the anti-inflammatory actions of immune cells [61,62]. This regulation of inflammation through NO is crucial in controlling neurodegeneration by suppressing inflammation in neuroinflammatory diseases [63]. Additionally, NO can be beneficial in the treatment of osteoarthritis by strongly inhibiting the expression of gelatinases like MMP-2 and MMP-9, which play a significant role in cartilage and bone destruction [64,65].

The potent inflammatory and MMPs inhibitory efficacy of SHP-47B was also observed in an animal model of rheumatoid arthritis induced by MIA (monosodium iodoacetate), which is known to induce rheumatoid arthritis as a glyceraldehyde-3-phosphate dehydrogenase inhibitor. The MIA-induced arthritis model in rats causes joint cartilage damage, functional impairment, and pain similar to human rheumatoid arthritis. Injecting MIA into the knee joint space of rats causes changes in the synovial membrane and surrounding tissues, increasing the load on the cartilage and causing continuous pain [35]. In this MIA-induced arthritis animal model, treatment with the SHP-47B reduced pain (joint arthritis index, hindlimb index, and gait analysis) induced by MIA and restored damage to the meniscus cartilage, effectively reducing the levels of inflammatory cytokines TNF-α and IL-6, inflammatory factor PGE2, MMP-2, and nitric oxide (NO) as confirmed by hematological analysis. The anti-inflammatory efficacy of SHP-47B was demonstrated through significant results in clinical index analysis and histological analysis of experimental animals. Specifically, a concentration-dependent alleviation of osteoarthritis was observed in the weight-bearing index and arthritis clinical index with SHP-47B treatment (50, 100, and 300 mg/kg).

In addition, the Micro CT-arthrography analysis of the meniscus, a crescent-shaped cartilage located between the knee joints that plays a role in joint protection, shock absorption, and assisting knee function, showed that the volume of the meniscus increased in a concentration-dependent manner with SHP-47B treatment (50, 100, and 300 mg/kg).

Finally, histological analysis of the left knee joint in each group was performed using hematoxylin and eosin (H&E) staining and Safranin-O fast green staining. Based on the pathophysiological observations of the disease model, treatment approaches can be divided into direct tumor removal, alleviation of disease symptoms, and disease progression inhibition through medication, as well as rehabilitation therapy. The purpose of our research findings seems to lie in identifying methods that can alleviate or suppress the progression of the disease [66]. Treatment of experimental animals with MIA resulted in significant loss of cartilage tissue (bone erosion) and infiltration of inflammatory cells in the tissue around the joint. In addition, inflammation invaded the cartilage along with synovial inflammation and caused widespread destruction of the bone tissue beneath the cartilage due to the loss of proteoglycans. However, treatment with SHP-47B at concentrations of 50, 100, and 300 mg/kg resulted in a concentration-dependent decrease in cartilage tissue loss and infiltration of inflammatory cells. In particular, it was confirmed that treatment with a high concentration (300 mg/kg) of SHP-47B suppressed osteoarthritis by reducing cartilage tissue loss and infiltration of inflammatory cells and restoring the proteoglycan layer.

Various natural products are being studied for their potential to improve osteoarthritis, but many of these products lack scientific evidence regarding quality control and efficacy. In this study, we investigated the anti-osteoarthritis efficacy of SHP-47B, a mixture of components *Kalopanax pictus* Nakai and *Achyranthes japonica* Nakai, rather than a single ingredient, similar to other existing literature.

Since inflammatory cytokines are known to be involved in the initiation and perpetuation of the osteoarthritis process, we treated LPS-induced RAW264.7 cells with SHP-47B to inhibit inflammation-related proteins. Additionally, we conducted in vivo experiments using an MIA-induced animal model to evaluate the anti-inflammatory cytokine inhibition and histological analysis, indicating the potential of SHP-47B as a functional material for improving osteoarthritis. However, most studies have focused on inflammatory cytokines, and further mechanistic research involving various protein analyses related to inflammatory cytokines should be conducted for a more detailed understanding.

## 5. Conclusions

The purpose of this study was to develop pharmaceuticals using natural substances, and we utilized two types of natural substances with known efficacy. Cell experiments and animal experiments were conducted, and, through various research outcomes, the efficacy of these substances against arthritis could be verified. However, to proceed with the development of pharmaceuticals, there are still many aspects that need to be confirmed. In particular, MAPK and NF-κB act as crucial signaling pathways within the cells, and understanding their activation, inhibition mechanisms, as well as the interactions of related proteins and genes, is essential. Moreover, through this understanding, we can determine the internal responses induced by external stimuli and unveil the relevance of disruptions in these signaling pathways to specific diseases.

To conduct mechanistic studies of MAPK and NF-κB, it is crucial to include appropriate negative control and positive control groups, ensuring consistent experimental repetition to validate the results. Moreover, employing diverse techniques to assess the activities of MAPK and NF-κB and confirm their interactions appears to be necessary.

By pursuing such an approach, conducting meticulous mechanistic studies of the MAPK and NF-κB signaling pathways, we can elucidate the mechanisms underlying the anti-inflammatory effects associated with arthritis. This can pave the way for the development of novel therapeutic interventions utilizing *Kalopanax pictus* Nakai and *Achyranthes japonica* Nakai extracts.

## Figures and Tables

**Table 1:** Effect of Herbal Complex on Acetic Acid-Induced Writhing Test in Mice (**A**). Effect of *Achyranthes japonica* Nakai and *Kalopanax pictus* Nakai Complex on Acetic Acid-Induced Writhing Test in Mice (**B**). Values with different superscript letters indicate significant differences within treatment groups at *p* < 0.05 by ANOVA and Duncan’s multiple range tests. Data are presented as the means ± standard errors (*n* = 5).

Group		Dose (mg/kg, P.O)	Average No. of Writhing	Decrease in Writhing	Inhibition (%)
Control	A	-	59.00 ± 7.23 ^de^	0.00 ^ab^	0 ^ab^
HPS-3C		100	54.33 ± 0.88 ^cde^	4.67 ^abc^	7.91 ^abc^
SHP-40		100	35.67 ± 8.19 ^bc^	23.33 ^cd^	39.55 ^cd^
SHP-41		100	43.00 ± 5.13 ^bcd^	16.00 ^bcd^	27.12 ^bcd^
SHP-42		100	47.00 ± 1.15 ^bcde^	12.00 ^abcd^	20.34 ^abcd^
SHP-43		100	15.33 ± 8.41 ^a^	43.67 ^e^	74.01 ^e^
SHP-44		100	58.67 ± 4.37 ^de^	0.33 ^ab^	0.56 ^ab^
SHP-45		100	68.33 ± 6.39 ^e^	−9.33 ^a^	-15.82 ^a^
SHP-46		100	54.33 ± 10.84 ^cde^	4.67 ^abc^	7.91 ^abc^
SHP-47		100	11.67 ± 7.22 ^a^	47.33 ^e^	80.23 ^e^
SHP-48		100	51.33 ± 6.36 ^cde^	7.67 ^abc^	12.99 ^abc^
Indomethacin		10	27.00 ± 3.21 ^ab^	32.00 ^de^	54.24 ^de^
SHP-47A	B	100	36.67 ± 0.88		
SHP-47B			30.33 ± 4.48		
SHP-47C			32.33 ± 1.20		
SHP-47D			43.00 ± 7.55	32.00 ^de^	54.24 ^de^

**Table 2:** Effects of SHP-47B Treatment on blood immune cells in MIA-induced arthritis experimental mice models. The data are expressed as the mean ± SD (*n* = 7), and different letters (d > c > b > a) indicate a significant difference at *p* < 0.05, as determined by Duncan’s multiple-range test.

Group	×10^3^ cell/μL				% of WBC		
	WBC	GRA	LYM	MID	GRA	LYM	MID
Normal	8.49 ± 0.48 ^b^	2.03 ± 0.18 ^c^	6.21 ± 0.51 ^abc^	0.23 ± 0.02 ^b^	22.63 ± 1.39 ^d^	70.67 ± 1.52 ^a^	2.46 ± 0.17
Control	7.73 ± 0.34 ^ab^	1.28 ± 0.05 ^a^	6.20 ± 0.30 ^abc^	0.17 ± 0.01 ^a^	15.21 ± 0.50 ^a^	80.10 ± 0.95 ^d^	2.16 ± 0.06
SPH-47B-50	8.27 ± 0.38 ^b^	1.51 ± 0.11 ^ab^	6.33 ± 0.32 ^bc^	0.20 ± 0.01 ^ab^	17.24 ± 1.33 ^ab^	75.40 ± 1.03 ^bc^	2.27 ± 0.15
SPH-47B-100	8.96 ± 0.56 ^b^	1.61 ± 0.12 ^ab^	7.08 ± 0.50 ^c^	0.20 ± 0.01 ^ab^	16.61 ± 0.71 ^ab^	77.69 ± 1.01 ^cd^	2.17 ± 0.13
SPH-47B-300	6.80 ± 0.31 ^a^	1.41 ± 0.10 ^ab^	5.14 ± 0.19 ^a^	0.17 ± 0.02 ^a^	19.37 ± 0.94 ^bc^	74.96 ± 0.96 ^bc^	2.27 ± 0.07
Celecoxib	7.73 ± 0.29 ^ab^	1.67 ± 0.05 ^b^	5.74 ± 0.27 ^ab^	0.19 ± 0.01 ^ab^	20.93 ± 0.84 ^cd^	73.61 ± 0.92 ^b^	2.44 ± 0.08

## Data Availability

Not applicable.

## Supplementary Materials

The following supporting information can be downloaded at: https://www.mdpi.com/article/10.3390/cimb45080404/s1, Figure S1: Effect of SHP-47 treatment on hematological analysis; Figure S2: Effects of SHP-47 treatment on weight of mice in MIA-induced Arthritis mice models; Table S1: Anti-body used in Western blot experiments.
