# Supplementary material for: The Effect of the Mixed Extract of Kalopanax pictus Nakai and Achyranthes japonica Nakai on the Improvement of Degenerative Osteoarthritis through Inflammation Inhibition in the Monosodium Iodoacetate-Induced Mouse Model
Source: Curr Issues Mol Biol. 2023 Aug 1;45(8):6395–414. doi: 10.3390/cimb45080404 (PMC10453891; doi:10.3390/cimb45080404)
Supplement: Supplementary file 1 [file cimb-45-00404-s001.zip › cimb-2479254-supplementary.pdf]

Table S1. Anti-body used in Western blot experiments.

Antibody	Source	Cat. No.	Manufacturer
ERK	Rabbit	#9102S	Cell Signaling
p-ERK	Rabbit	#9101S	Cell Signaling
JNK	Rabbit	#9252S	Cell Signaling
p-JNK	Rabbit	#4671S	Cell Signaling
p38	Rabbit	#9212S	Cell Signaling
p-p38	Rabbit	#4511S	Cell Signaling
NF-kB	Rabbit	#8242S	Cell Signaling
p-NF-kB	Rabbit	#3033S	Cell Signaling
iNOS	Rabbit	ab178945	Abcam
β-actin	Mouse	sc-47778	Santa Cruze
anti-Rb-HRP	Mouse	sc-2357	Santa Cruze
anti-ms-HRP	Goat	sc-516102	Santa Cruze

As a result of analyzing inflammatory cytokines and MMPs in the culture of LPS-induced RAW 264.7 using ELISA kit, it was confirmed that the expression of TNF-α, IL-6, PGE2, MMP-2 and MMP-9 was significantly increased in the LPS treatment group.

In the case of the SHP-47B treatment group, TNF-α, IL-6, PGE2, MMP-2 and MMP-9 were inhibited in a concentration-dependent manner, and in the MMP-2 result, the results were similar to those of the control group from the SHP-47B 500 ug/mL concentration. showed

Through these results, it was thought that SHP-47 would be able to derive significant results in subsequent arthritic animal experiments.

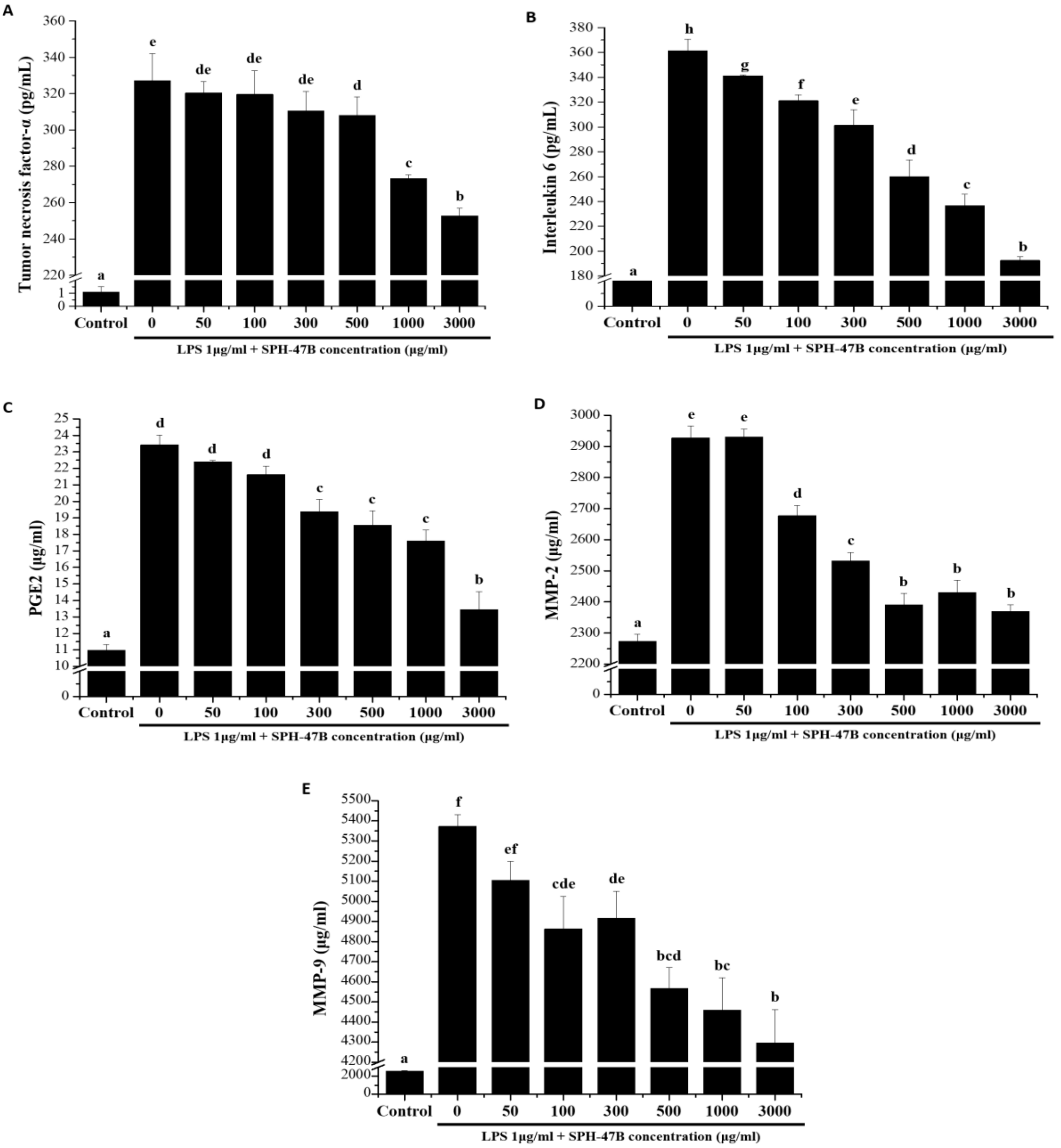

Figure S1. Effect of SHP-47 treatment on hematological analysis, (A) tumor necrosis factor-α (TNF-α), (B) interleukin-6 (IL-6), (C) prostaglandin E2 (PGE2), (D) matrix metalloproteinase-2 (MMP-2) and (E) matrix metalloproteinase-9 (MMP-9) in a mice model of arthritis induced by MIA. The data are expressed as the mean ± SD (n = 7), and different letters (h>g>f>e>d > c > b > a) indicate a significant difference at $p < 0.05$, as determined by Duncan's multiple-range test.

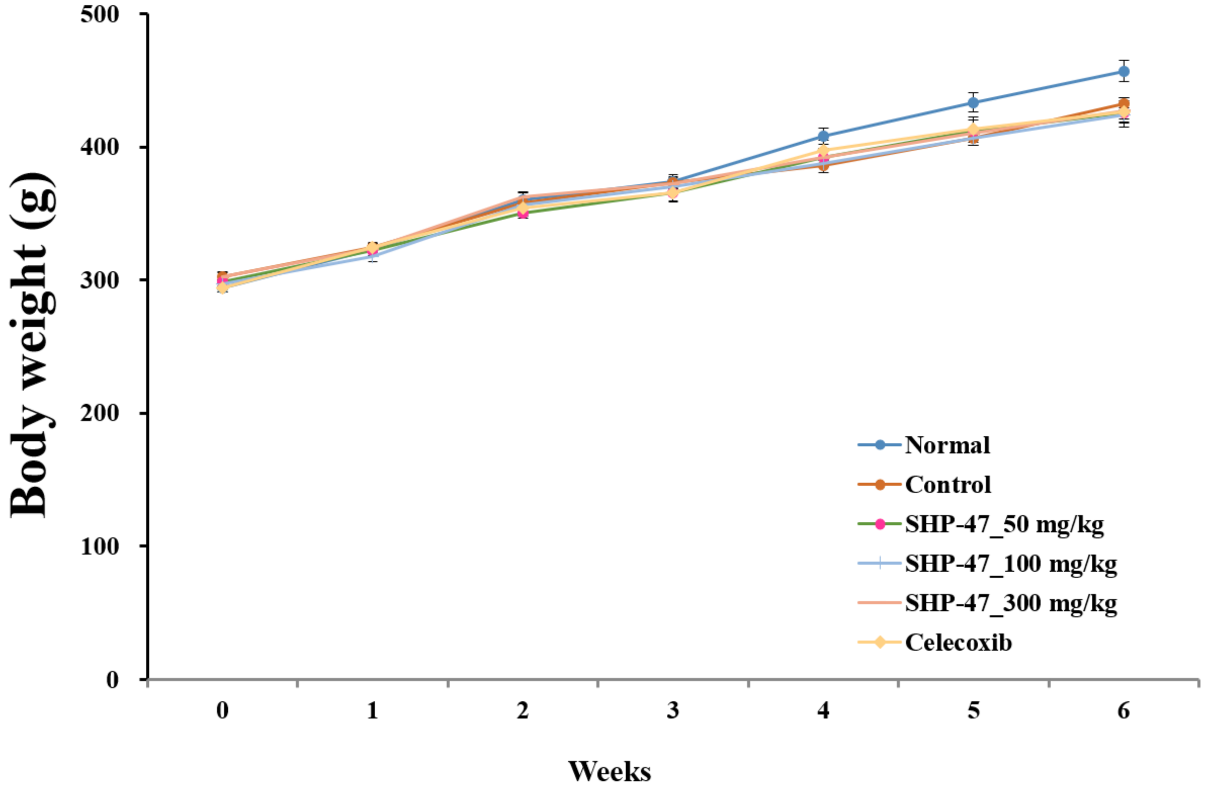

Figure S2. Effects of SHP-47 treatment on weight of mice in MIA-induced Arthritis mice models. N : Normal, C : Control, L : SHP-47 50 mg/kg, M : SHP-47 100 mg/kg, H : SHP-47 300 mg/kg, P : celecoxib. The data are expressed as the mean ± SD (n = 7), and different letters (b > a) indicate a significant difference at $p < 0.05$, as determined by Duncan's multiple-range test.
